# BAD sensitizes breast cancer cells to docetaxel with increased mitotic arrest and necroptosis

**DOI:** 10.1038/s41598-019-57282-1

**Published:** 2020-01-15

**Authors:** Jasdeep Mann, Ning Yang, Rachel Montpetit, Raven Kirschenman, Hélène Lemieux, Ing Swie Goping

**Affiliations:** 1 Department of Biochemistry, University of Alberta, Edmonton, Alberta T6G 2H7 Canada; 2 Department of Medicine, University of Alberta, Edmonton, Alberta T6G 2H7 Canada; 3 Faculty Saint-Jean, University of Alberta, Edmonton, Alberta T6G 2H7 Canada; 4 Department of Oncology, University of Alberta, Edmonton, Alberta T6G 2H7 Canada

**Keywords:** Cancer therapeutic resistance, Chemotherapy, Tumour biomarkers, Breast cancer

## Abstract

Breast cancer patients are commonly treated with taxane (e.g. docetaxel) chemotherapy, despite poor outcomes and eventual disease relapse. We previously identified the Bcl-2-associated death promoter (BAD) as a prognostic indicator of good outcome in taxane-treated breast cancer patients. We also demonstrated that BAD expression in human breast carcinoma cells generated larger tumors in mouse xenograft models. These paradoxical results suggest that BAD-expressing tumors are differentially sensitive to taxane treatment. We validated this here and show that docetaxel therapy preferentially reduced growth of BAD-expressing xenograft tumors. We next explored the cellular mechanism whereby BAD sensitizes cells to docetaxel. Taxanes are microtubule inhibiting agents that cause cell cycle arrest in mitosis whereupon the cells either die in mitosis or aberrantly exit (mitotic slippage) and survive as polyploid cells. In response to docetaxel, BAD-expressing cells had lengthened mitotic arrest with a higher proportion of cells undergoing death in mitosis with decreased mitotic slippage. Death in mitosis was non-apoptotic and not dependent on Bcl-XL interaction or caspase activation. Instead, cell death was necroptotic, and dependent on ROS. These results suggest that BAD is prognostic for favourable outcome in response to taxane chemotherapy by enhancing necroptotic cell death and inhibiting the production of potentially chemoresistant polyploid cells.

## Introduction

Triple-negative breast cancer patients receive taxane chemotherapy, such as docetaxel (Taxotere®), as standard first-line treatment despite an overall poor prognosis, high rate of relapse, and adverse effects^1^. While multiple causes of cellular taxane resistance are known, these have not yet provided clinical markers to guide taxane therapy decisions^2–4^. Understanding the molecular mechanisms that mediate outcome to taxane therapy may identify predictive biomarkers and novel therapeutic targets. The Bcl-2 family member BAD (Bcl-2-associated death promoter) is a prognostic indicator for good clinical outcome of taxane-treated breast cancer patients^5^. BAD modulates breast cancer cell proliferation and tumor progression by regulating cell cycle progression^6,7^. Thus, understanding how BAD better predicts patient outcome could aid in understanding docetaxel chemoresistance.

Taxanes are anti-mitotic drugs that perturb microtubule dynamics, leading to chronic activation of the spindle assembly checkpoint and inhibition of the anaphase promoting complex that delays the degradation of cyclin B1 and inhibits mitotic exit^8^. Ideally, this aberrant mitotic arrest initiates cell death in mitosis by facilitating the accumulation of a caspase-dependent death signal^9^. Often, however, cells degrade sufficient cyclin B1 prior to full activation of apoptotic caspases, and cells slip out of mitosis in the absence of cytokinesis and enter G1 as polyploid cells. These polyploid cells have differential fates of G1 arrest, post-mitotic death, or continued cell cycle progression^10,11^. The survival and expansion of these polyploid cells is postulated to generate aggressive clones that are resistant to therapy^12,13^.

Programmed necrosis, termed necroptosis, is a form of caspase-independent cell death that is activated in response to many anticancer drugs^14^. Cells can activate necroptosis in the absence of functional apoptosis^15^. The cell morphology of necroptotic death is similar to that of necrosis, as it includes loss of plasma membrane integrity, mitochondrial dysfunction, oxidative stress, and absence of nuclear fragmentation^16^. Recent evidence suggests necroptosis can be exploited for cancer therapy, in particular, for apoptosis-resistant cancers^17^. Taxane treatment can promote necroptosis, although the mechanism remains unclear^18^.

In the current study, we demonstrated that BAD increases sensitivity of breast cancer tumor xenografts to docetaxel treatment *in vivo*. BAD-expressing cells prolonged mitotic arrest, and enhanced cell death. Cell death was not dependent on caspases or Bcl-XL indicating a non-apoptotic pathway. Instead, cell death had morphological hallmarks of necrosis and was dependent on reactive oxygen species (ROS) and the necroptotic kinase MLKL. Thus, we identified a novel role for BAD in enhancing necroptosis during taxane-induced mitotic arrest. Our results provide a potential cellular mechanism wherein BAD is prognostic for clinical docetaxel chemotherapy.

## Results

### BAD increases sensitivity of breast cancer cells to docetaxel

**Figure 1:**
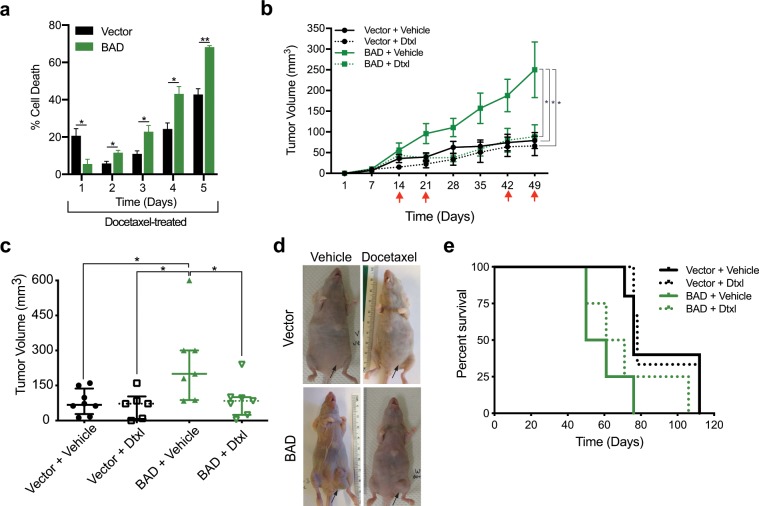
BAD increases sensitivity to docetaxel. (**a**) MDA-MB-231 cells expressing vector or BAD were treated with 125 nM docetaxel for 5 days. Cells were stained with Annexin V-647 and PI and analyzed via flow cytometry daily. Cell death in control group were subtracted from the docetaxel treated group. Annexin V+/PI+ population is depicted. Student’s *t*-test; n = 3. (**b**) MDA-MB-231 cells expressing vector or BAD were injected into the mammary fat pads of Taconic nude mice. Red arrows indicate docetaxel or vehicle injection time points. Tumor volume was measured weekly. One-way ANOVA with Dunnett’s post-hoc test; Vector + vehicle = 8, vector + docetaxel = 6, BAD + vehicle = 7, BAD + docetaxel = 7. (**c**) Scatter plot of tumor volume at day 49. (**d**) Representative images of tumors in the mammary gland of nude mice. Arrows indicate tumor location. (**e**) Kaplan-Meier survival curve of mice treated with vehicle or docetaxel.

We had previously shown BAD-dependent taxane sensitivity in the breast carcinoma cell lines MCF-7 (luminal B), SKBR-3 (HER2) and MDA-MB-468 (TNBC)^5^. To study the structure/function relationship of BAD in docetaxel-treated breast cancer cells, we utilized MDA-MB-231 cells stably expressing ectopic BAD^7^. We performed a longitudinal cell death assay over 5 days of docetaxel treatment to determine docetaxel sensitivity (Fig. 1a). At the earliest measured time point BAD protected cells from docetaxel-induced cell death. This was transient, however, as with increasing time BAD sensitized cells to docetaxel-toxicity. To examine the *in vivo* relevance of these effects, we performed orthotopic mammary fat pad xenografts in nude mice. Mice were treated with docetaxel on the days indicated by the red arrows (Fig. 1b) and tumor volume was measured. Similar to what we had reported previously, BAD tumors grew significantly larger than vector tumors due to increased cell proliferation and survival signalling^7^. Tumor growth of BAD expressing cells was significantly decreased in response to docetaxel treatment (Fig. 1c,d). On the other hand, there was no change in tumor size in docetaxel-treated vector control tumors. Additionally, overall survival of mice with BAD tumors treated with docetaxel was increased relative to untreated BAD tumors (Fig. 1e). Altogether, these results indicate BAD expression increases tumor volume, however, these cells are more sensitive to docetaxel treatment with enhanced cell death and decreased tumor size.

### BAD increases length in docetaxel-mediated mitotic arrest to promote cell death over mitotic slippage

**Figure 2:**
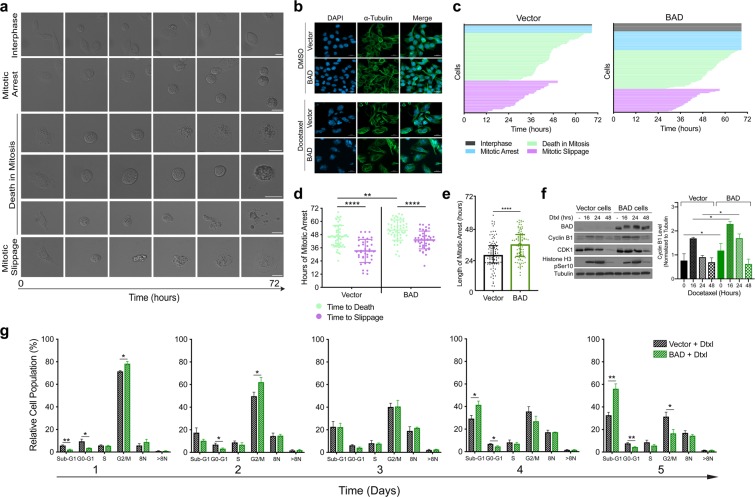
BAD increases length in mitotic arrest with docetaxel treatment. (**a**) Representative cell fates of MDA-MB-231 cells expressing vector or BAD treated with 125 nM docetaxel for 72 hours. Scale bar = 20 μM. (**b**) Immunofluorescence images taken 48 hours after docetaxel treatment and stained with DAPI and α-Tubulin. Scale bar = 20 μM. (**c**) Cell fates of cells treated with docetaxel for 72 hours. Each horizontal line represents an individual cell. Line endpoint represents the time at which the indicated cell fate occurred. (**d**) Scatter-plot representation of individual cell which either slipped or died in mitosis, and the corresponding hours of mitotic arrest. Median and interquartile range are shown. Mann-Whitney statistical test. (**e**) Length of mitotic arrest for all cell fates. Mann-Whitney statistical test. (**f**) Cells were treated with 125 nM docetaxel for 16, 24, or 48 hours. DMSO control treatment was for 48 hours. Right: Quantification of cyclin B1 levels normalized to tubulin. Student’s *t*-test; n = 3. (**g**) Cell cycle phases of docetaxel treated cells were analyzed daily for 5 days with PI staining and flow cytometry. Student’s *t*-test; n = 3.

Docetaxel binds to tubulin and disrupts microtubule dynamics, induces prolonged mitotic arrest, and can lead to cell death in mitosis^19^. Cells that do not die in mitosis ‘slip’ out of mitosis and re-enter G1 without cytokinesis. This phenomenon is referred to as ‘mitotic slippage’^20^. To understand how BAD sensitized cells to docetaxel, we used time-lapse live imaging to characterize cell morphology in response to docetaxel. Representative cell fates of docetaxel-treated cells are shown (Fig. 2a). We defined death in mitosis as cells with mitotic morphology that eventually ceased all cell movement. This cell death had a non-apoptotic morphology, indicated by cell swelling, granulation of the cytoplasm, and lack of cellular blebbing. Mitotic slipped cells were defined as cells with mitotic morphology that then transitioned to become flat adherent cells. These slipped cells remained viable and maintained subcellular movements. Mitotic slippage at imaging endpoint was confirmed by DAPI-staining, which revealed large multinucleated cells (Fig. 2b), typical of cells that have undergone mitotic exit in the absence of cytokinesis^21^.

To quantify differences in cell fates in vector versus BAD cells, we generated cell fate maps over 72 hours of drug treatment (Fig. 2c). The bar length indicates the time each cell spent in mitotic arrest, and the bar color indicates the subsequent cell fate until the experimental endpoint. Cells that entered mitosis (Fig. 2c) either remained in mitosis (blue), died in mitosis (green) or slipped out of mitotic arrest (purple). The average length a cell spent in mitotic arrest that culminated in death in mitosis was longer than for the cells that underwent slippage, (Fig. 2d), supporting a model of competing pathways between cell death and mitotic exit^22,23^. Additionally, BAD cells showed significantly longer times in mitotic arrest than vector control cells, irrespective of cell fate (Fig. 2e). Degradation of cyclin B1 is critical for determining cell fate, as the premature attenuation of cyclin B1 prior to accumulation of a sufficient cell death signal ultimately leads to mitotic slippage^24^. BAD cells retained higher levels of cyclin B1, suggesting that enhanced cyclin B1 stability lengthened mitotic arrest thus enabling the cells to accumulate a cell death signal (Fig. 2f). In support of this, DNA content-based cell cycle analysis revealed a greater proportion of BAD cells in mitotic arrest (G2/M; days 1–2), that then is followed by a higher amount of cell death (sub-G1 population; days 4–5) (Fig. 2g). Taken together, these results reveal BAD increases length in mitotic arrest upon docetaxel treatment in association with inhibited cyclin B1 degradation. This may allow the cells to accumulate a greater cell death signal, favouring cell death in mitosis versus mitotic slippage. These data therefore suggest BAD sensitizes cells to docetaxel by facilitating mitotic arrest-dependent non-apoptotic cell death.

### The BAD and Bcl-XL interaction is dispensable for docetaxel cell death

**Figure 3:**
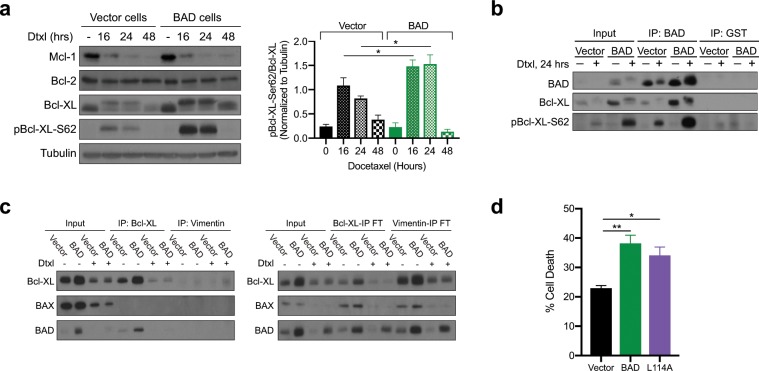
BAD and Bcl-XL binding is not required for docetaxel killing. (**a**) Left: MDA-MB-231 cells stably expressing vector or BAD were treated with 125 nM docetaxel for the indicated time points. Control is DMSO for 48 hours. Right: Quantification of protein band density for pBcl-XL-Ser62 over total Bcl-XL. Student’s *t*-test; n = 3. (**b**) Immunoprecipitation (IP) of BAD antibody after 24 hours of 125 nM docetaxel treatment. GST antibody was used as a negative control. (**c**) Immunoprecipitation with Bcl-XL antibody after 125 nM docetaxel for 5 days. Vimentin antibody was used as a negative control. Right: The flow throughs (FT) from the IPs were retained and subjected to western blot. (**d**) Annexin V+/PI+ staining and flow cytometry analysis of 125 nM docetaxel treated cells after 5 days. One-way ANOVA with Dunnett’s post-hoc test; n = 5.

The anti-apoptotic Bcl-2 family members Mcl-1, Bcl-2, and Bcl-XL regulate death in mitosis and mitotic slippage. Mcl-1 degradation is required for death in mitosis^25^. Additionally, phosphorylation of Bcl-2, and Bcl-XL by CDK1 upon taxane treatment inactivates these anti-apoptotic proteins and enables mitotic death^26,27^, whereas lack of Bcl-XL phosphorylation is associated with mitotic slippage and cell survival^26^. Thus, we examined Mcl-1, Bcl-2, and Bcl-XL after docetaxel addition to determine if BAD cells elevated death in mitosis due to increased degradation or phosphorylation of these anti-apoptotic Bcl-2 family members. We found no difference in Mcl-1 and Bcl-2 levels between vector and BAD cells (Fig. 3a). However, Bcl-XL levels were higher with more phosphorylation on Ser62 in BAD cells compared to vector cells (Fig. 3a), confirming a higher proportion of BAD-expressing cells with active CDK1 in mitotic arrest. Furthermore, phosphorylation of Ser62 attenuates the anti-apoptotic activity of Bcl-XL by inhibiting binding to BAX^28^, suggesting cells are sensitized to undergo apoptosis. Since BAD binds to Bcl-XL to displace BAX and stimulate cell death^29^, we tested whether the BAD and Bcl-XL interaction was necessary for docetaxel-mediated cell death. Immunoprecipitation of BAD protein at 24 hours of docetaxel treatment revealed a strong interaction between BAD and phospho-Bcl-XL (Ser62) (Fig. 3b). Surprisingly, we did not see a Bcl-XL and BAX interaction under any conditions (Fig. 3c), which was inconsistent with an apoptotic “primed to die” phenotype. Therefore, we functionally tested the requirement of the BAD and Bcl-XL interaction for docetaxel-mediated cell death using a cell line expressing BAD-L114A that abrogates BAD:Bcl-XL binding^30^. The BAD L114A mutant did not attenuate docetaxel cell death, indicating the BAD and Bcl-XL interaction is not required for cell death (Fig. 3d). Thus, these results indicate that BAD-potentiation of docetaxel sensitivity is not dependent on binding to Bcl-XL.

### Docetaxel can induce necroptotic cell death

**Figure 4:**
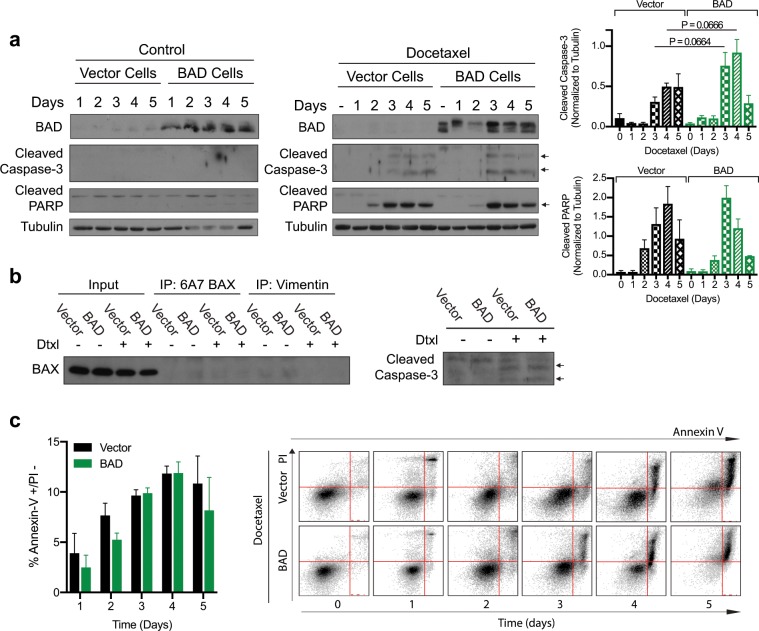
Docetaxel is killing the cells through a non-apoptotic mechanism. (**a**) MDA-MB-231 cells stably expressing vector or BAD were treated with DMSO control (left) or 125 nM docetaxel (middle) and subjected to western blot. Right: Protein band quantification was performed. Student’s *t*-test; no statistical significance. (**b**) Cells were treated with 125 nM docetaxel for 3 days prior to immunoprecipitation (IP) with 6A7 BAX antibody. Vimentin antibody was used as a negative control. Input lanes were also probed with cleaved caspase-3 antibody (right). (**c**) Cells were treated with 125 nM docetaxel for 5 days and stained with Annexin V-647 and PI daily and analyzed via flow cytometry. The Annexin V+/PI− population is graphed. Student’s *t*-test; no statistical significance between vector and BAD. Right: Dot plots from flow cytometric analysis. Annexin V positive cells are on the x-axis, PI positive cells are on the y-axis. Time, in days, is increasing to the right.

The observation that Bcl-XL does not regulate the ability of BAD to enhance docetaxel-induced cell death, suggests that BAD modulates a non-apoptotic mechanism. To assess apoptosis, we examined the apoptotic markers of cleaved caspase-3, cleaved PARP and BAX activation. Docetaxel treatment induced minimal caspase cleavage and detectable PARP cleavage that was similar between vector and BAD-expressing cells (Fig. 4a). BAX activation was not induced by docetaxel treatment (Fig. 4b). On the other hand, these markers were positive for apoptosis induced by a known apoptotic inducer, staurosporine (Supplementary Fig. 1)^31^. Therefore, despite having a competent apoptotic machinery, apoptosis is minimally activated with docetaxel. Instead, the majority of cells appeared necrotic with compromised plasma membranes (Annexin-V positive/PI positive) (Fig. 1a). In contrast, apoptotic inducers trigger initial phosphatidylserine externalization followed by secondary necrosis (Annexin-V positive/PI negative) (Fig. 4c and Supplementary Fig. 1). Thus, docetaxel caused concurrent plasma membrane damage and phosphatidylserine exposure, consistent with necrotic-like cell death.

**Figure 5:**
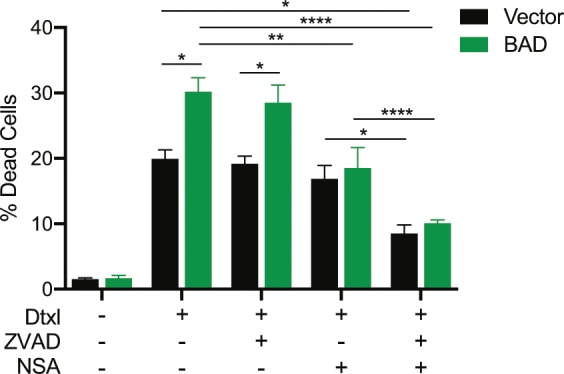
Docetaxel is killing the cells through a necroptotic mechanism. MDA-MB-231 cells stably expressing vector or BAD were treated with 125 nM docetaxel (Dtxl), 20 μM Z-VAD-FMK (ZVAD), or 5 μM necrosulfonamide (NSA) for 5 days. Cells were stained with Annexin V-647 and PI and analyzed via flow cytometry. The Annexin V+/PI+ population is represented in a bar graph. Two-way ANOVA with Tukey’s post-hoc test; n = 3.

Necroptosis is a regulated form of necrotic cell death that compensates when the apoptotic pathway is blocked^32^. The necrosome is the core executioner complex that consists of RIP3 oligomers and MLKL and is activated in a RIP1-dependent and independent manner^32,33^. Docetaxel has previously been shown to initiate both necrosis and apoptosis in MDA-MB-231 cells^34^, so we assessed whether necroptosis was triggered in our studies. Inhibition of caspases did not attenuate cell death (Fig. 5), consistent with necroptotic signaling. Inhibition of necroptosis with the MLKL inhibitor necrosulfonamide^35^ significantly decreased cell death in BAD cells only, indicating that BAD stimulates docetaxel-induced necroptosis. The addition of both a pan-caspase inhibitor, z-VAD-FMK, and necrosulfonamide significantly reduced cell death in both vector and BAD cells, confirming one mechanism of cell death might be activated when the other is compromised. Therefore, BAD stimulates docetaxel-induced cell death through necroptosis.

### Oxidative phosphorylation regulates docetaxel cell death

**Figure 6:**
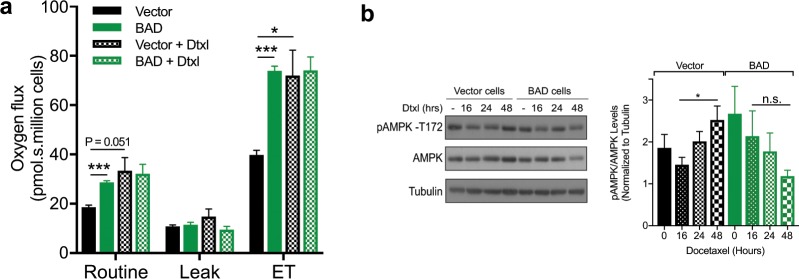
BAD-mediated mitotic arrest is downstream of AMPK. (**a**) MDA-MB-231 cells stably expressing vector or BAD were treated with 125 nM docetaxel for 48 hours prior to high-resolution respirometry. One-way ANOVA with Dunnett’s post-hoc test; n = 3. (**b**) Cells were treated with 125 nM docetaxel for 16, 24, or 48 hours. DMSO control treatment was for 48 hours. Right: Quantification of pAMPK-Thr172 levels over AMPK total protein were normalized to tubulin. One-way ANOVA with Dunnett’s post-hoc test; n = 3.

We have previously shown that BAD increases mitochondrial metabolism to promote cell survival^7^. AMP-activated protein kinase (AMPK), a cell energy sensor, boosts mitochondrial respiration during low energy status in mitosis and inhibits ROS-mediated necroptosis^36,37^. We therefore measured whether BAD expression altered oxygen consumption rate and AMPK activation upon docetaxel treatment. As expected, BAD expression increased oxygen consumption^7^ and this was unaffected by docetaxel treatment (Fig. 6a). Correspondingly, levels of active phosphorylated AMPK did not change in response to docetaxel. On the other hand, in vector control cells, docetaxel elevated the oxygen flux to reach similar levels to BAD-expressing cells (Fig. 6a). In line with this, AMPK activation also increased in response to docetaxel, suggesting that in vector cells, AMPK could attenuate necroptosis (Fig. 6b). Therefore, these data suggest BAD maintains adequate energy levels during mitotic arrest to circumvent AMPK activation and facilitate increased necroptosis.

### BAD requires reactive oxygen species for docetaxel induced cell death

**Figure 7:**
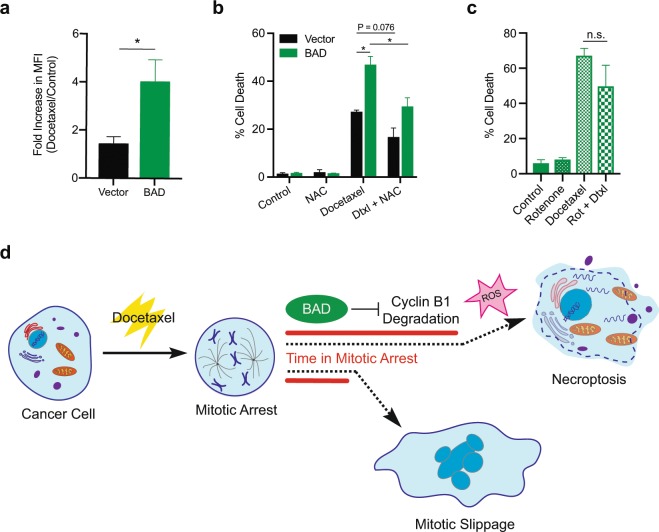
BAD requires ROS for docetaxel cell death. (**a**) MDA-MB-231 cells stably expressing vector or BAD were treated with 125 nM docetaxel or DMSO control for 48 hours prior to staining with CM-H_2_DCFDA to measure reactive oxygen species (ROS). Mean fluorescence intensity (MFI) was measured via flow cytometry. Fold increase of docetaxel/control is graphed. Student’s *t*-test; n = 3. (**b**) MDA-MB-231 cells stably expressing vector or BAD were treated with 125 nM docetaxel or 10 mM N-acetyl cysteine (NAC) for 5 days. Annexin V+/PI+ staining flow cytometry analysis of cells is graphed. Two-way ANOVA with Tukey’s post-hoc test; n = 3. (**c**) MDA-MB-231 cells expressing BAD were treated with 125 nM docetaxel or 50 nM rotenone for 5 days. Annexin V+/PI+ staining flow cytometry analysis of cells is graphed. Student’s *t*-test; n = 3. (**d**) Schematic representation of docetaxel-mediated cell death. BAD increases length in mitotic arrest by inhibiting cyclin B1 degradation, leading to a ROS-dependent necroptotic cell death.

BAD promotes cell survival and mitochondrial metabolism by stimulating complex I activity of the electron transport chain^7^, which is a main site of cellular ROS production^38^. Reactive oxygen species (ROS) are metabolic by-products of mitochondrial respiration and are required for maintaining redox-homeostasis of cancer cells^39^. Additionally, ROS has been shown to facilitate necroptosis^40,41^. BAD-expressing cells significantly increased ROS levels in response to docetaxel relative to vector control cells (Fig. 7a). To determine if BAD-enhanced necroptosis was dependent on ROS, we measured cell death in the presence of the ROS scavenger N-acetyl cysteine (NAC). Cell death was significantly attenuated only in docetaxel-treated BAD cells with NAC (Fig. 7b). We further examined whether ROS-dependent cell death in BAD cells was dependent on complex I. Inhibition of complex I by rotenone did not attenuate docetaxel cell death in BAD cells (Fig. 7c). Thus, these data suggest BAD confers docetaxel cell death that is dependent on ROS but does not require complex I activity. In conclusion, BAD prolongs docetaxel-mediated mitotic arrest to promote ROS-dependent necroptotic cell death (Fig. 7d).

## Discussion

In the current study, we show that BAD sensitizes cells to docetaxel treatment *in vitro* and *in vivo*. Docetaxel treatment did not significantly trigger apoptotic cell death and instead displayed necrotic cell death morphologies. Inhibition of the necroptosis executioner, MLKL, attenuated cell death, indicating that BAD enhanced docetaxel toxicity via necroptosis. BAD enhanced death in mitosis in association with increased length in mitotic arrest and elevated cyclin B1. Thus, BAD enhances docetaxel sensitivity by facilitating longer mitotic arrest and activating necroptotic cell death in mitosis.

Our previous results reveal high BAD protein levels are associated with a 3.7-fold increased probability of overall survival of primary breast cancer patients treated with taxane^5^. BAD stimulates cell cycle progression leading to increased breast cancer cell number and tumor growth^7^. Similarly, ectopic BAD expression increased prostate cancer cell number and tumor growth^42^. The present data validated BAD-dependent tumor growth in a mouse model and demonstrated that this increased tumor growth is sensitive to docetaxel. Docetaxel is an anti-mitotic drug that targets actively proliferating cells by stabilizing microtubules and inducing cell death^43^. Thus, docetaxel may target BAD cells more effectively due to their increased proliferation.

Necrosis is an unregulated form of cell death, while necroptosis is programmed necrosis characterized by the activation of the RIP3-dependent pathway^44^. Necroptosis is activated in response to many anticancer drugs and contributes to their cytotoxicity^45^. TNF was the first documented inducer of the necroptotic pathway^46^. In response to TNF, overactivation of PARP1 by ROS-mediated DNA damage was shown to cause necrosis^47^. More recently, TNF-induced necroptosis and PARP-1-mediated necrosis have been established as two distinct pathways to programmed necrosis^48^. Our results showed no difference in PARP cleavage between vector and BAD cells in response to docetaxel, suggesting that BAD did not specifically stimulate PARP-mediated necroptosis. Inhibiting apoptotic caspases with zVAD-FMK alone did not inhibit docetaxel-induced cell death, similar to previous reports by others with MDA-MB-231 cells^49^. Therefore, apoptosis is not a major cell death pathway for docetaxel in these triple-negative breast carcinoma cells. Recently, necroptosis activation has been shown to overcome chemotherapy resistance^50^. Aldehyde dehydrogenase inhibitors kill ovarian cancer stem cells by activating necroptosis, in part, by the induction of mitochondrial uncoupling proteins and reduction in mitochondrial oxidative phosphorylation^51^. In line with this, another group showed that docetaxel-resistant prostate cancer cells induced a shift from glycolysis to oxidative phosphorylation to confer a survival advantage^52^. In our model system, we saw a similar increase in mitochondrial metabolism in vector cells exposed to docetaxel, suggesting this was associated with a higher resistance to cell death than BAD cells.

BAD-enhanced docetaxel-mediated cell death was dependent on ROS. Impaired oxidative phosphorylation can lead to loss of inner transmembrane potential, reduction of ATP, and production of mitochondrial ROS^53^. However, oxidative phosphorylation was not impaired in docetaxel-treated BAD cells. Additionally, ROS levels peak in G2 and mitosis and can cause oxidative damage^54^. BAD increased length in docetaxel-mediated mitotic arrest, suggesting that increased ROS were a result of extended mitosis. Chemotherapeutic agents generate ROS in cancer cells to push ROS levels over a threshold to induce cell death^39^. Additionally, ROS can activate necroptosis in certain cell types^55^. Therefore, we hypothesize BAD-mediated prolonged mitotic arrest alters redox homeostasis during taxane treatment to cause cell death.

Docetaxel has been a standard chemotherapeutic regimen both alone and in combination for many different cancers^56^. Although docetaxel prolongs overall and disease-free survival, a significant number of patients eventually acquire chemoresistance and succumb to their disease. Polyploidization is a key factor in resistance and relapse of docetaxel therapy, and can arise following docetaxel-mediated mitotic arrest^57^. Polyploid cells are large, multinucleated cells that aberrantly exited mitosis without undergoing cytokinesis, through a process known as ‘mitotic slippage’^12^. Polyploid cells are characterized by excessive centrosomes, with unstable chromosomes, and are highly resistant to chemotherapy^13^. Slipped polyploid cells can continue to cycle, or emerge from chemotherapy-induced senescence with stem cell-like features and display a more aggressive phenotype^58,59^. In support of this, prostate cancer cells treated with docetaxel generated a population of ‘slipped’ cells, of which a small percentage survived and gave rise to a chemoresistant and cancer stem cell positive population^60^. We observed that BAD promotes death in mitosis over mitotic slippage. BAD cells had a significantly longer mitotic arrest phase and underwent significantly more mitotic necroptosis. These data suggest that this longer mitotic arrest allowed the cell death signal to accrue leading to a cell fate of death over cyclin B1 degradation-mediated slippage^23^. Thus, in response to taxane chemotherapy, BAD high patient tumors may undergo greater cell death with less mitotic slippage, and decreased capacity to evolve chemoresistance and cancer stem-like properties, in line with the increased overall survival of these patients.

In summary, BAD increases cell death in response to docetaxel treatment. We have shown BAD expression increases length in mitotic arrest, with more death in mitosis and less mitotic slippage and polyploidy. Additionally, this cell death is necroptotic and dependent on reactive oxygen species. Understanding the mechanism of docetaxel cell death will aid in understanding BAD’s function as a prognostic biomarker in breast cancer. This may guide future studies examining whether BAD predicts patient response to taxane therapy and may suggest non-taxane chemotherapy for breast cancer patients with low BAD levels.

## Materials and Methods

### Cell culture and reagents

MDA-MB-231 cells were from ATCC. Cells were cultured in RPMI 1640 medium (Life Technologies) with 10% FBS as previously described^5^. Ectopic BAD expression and stable cell line generation was as before^7^. Cell lines were routinely tested for mycoplasma contamination and passaged a maximum of 25 times. Z-VAD-FMK was from R&D systems, and necrosulfonamide was from Calbiochem.

### Flow cytometry analysis of cell death and cell cycle

Cells were incubated with 125 nM docetaxel (Sigma-Aldrich) or 2.5 μM staurosporine (Sigma-Aldrich) for the indicated times. Cells were harvested and washed twice with 1 X PBS prior to incubation with Annexin-V 647 (Invitrogen) in 1 X Annexin V binding buffer (1:20 dilution) for 15 minutes at room temperature in the dark, according to the manufacturer’s instructions. Cells were spun down to remove Annexin-V prior to addition of 20 μg/ml propidium iodide for 5 minutes (Life Technologies). Fluorescence was measured on the FL-4 and FL-2 channels with the BD Accuri™ C6 flow cytometer. To measure cell cycle, cells were fixed overnight in 70% ethanol and permeabilized with 0.25% Triton X-100 (Sigma-Aldrich) following addition of 10 μg/ml RNase A (Sigma-Aldrich) and 20 μg/ml PI.

### Mouse studies

Animal procedures were performed in accordance with the guidelines and regulations set forth by the Canadian Council on Animal Care and approved by the University of Alberta Health Sciences 2 Animal Care and Use Committee (Protocol # AUP00000386). 3 × 10^6^ cells were injected into the left mammary gland (#4) of nude mice (CrTac:NCr-*Foxn1*^*nu*^, Taconic) in 100 μl of a 1:1 matrigel/media mix. RPMI 1640 medium with no supplemental serum or antibiotic and Corning® Matrigel® Basement Membrane Matrix was used. Docetaxel (10 mg/kg) was administered via intraperitoneal injection on days as indicated. Animals were monitored weekly and sacrificed when tumors reached 20 mm in diameter. Tumors were collected and tumor volume (mm^3^) was calculated with the formula of (length × width × height)/2. Hematoxylin and eosin staining was performed as previously described^7^.

### Protein analysis

Protocols for immunoblotting and co-immunoprecipitation (IP) were as previously described^5^. Antibodies were from the following sources: Cyclin B1, CDK1, phospho-histone H3 Ser10, pAMPK-Thr172, AMPK, Mcl-1, Bcl-2, Bcl-XL, pBcl-XL-Ser62 and cleaved PARP were from Cell Signaling Technologies. BAD, tubulin and BAX conformational antibody clone 6A7 were from Sigma-Aldrich. BAX was from Santa Cruz Biotechnology, vimentin was from Abcam, and cleaved caspase-3 was from Enzo Life Sciences.

### Live-cell imaging

Cells were plated on Nunc™ Lab-Tek™ chambered coverglass prior to 125 nM docetaxel addition. A layer of mineral oil was layered over the medium to prevent evaporation. Cells were enclosed in a live-cell chamber with regulated temperature, humidity, and pH. Images were taken every 12 minutes up to 72 hours using a Zeiss AxioObserver Z1 Microscope, 20x objective lens.

### Immunofluorescence

Immunofluorescence was performed as previously described^7^.

### Reactive oxygen species

Cells were grown in 125 nM docetaxel or DMSO control for 48 hours prior to wash in Hank’s Balanced Salt Solution (ThermoFisher) followed by staining in 1.5 μM chloromethyl-2′, 7′-dichlorodihydrofluorescein diacetate (CM-H_2_DCFDA) (Invitrogen) for 45 minutes at 37 °C in the dark. Mean fluorescence intensity (MFI) was measured in the FL-1 channel using flow cytometry.

### High-resolution respirometry

High-resolution respirometry was performed as previously described^61^. Briefly, respiration of intact cells (1 × 10^6^ cells/mL) was measured in RPMI 1640 culture medium (10% fetal calf serum) under cellular routine conditions (ROUTINE). After inhibition of ATP-synthase with 2 μg/mL oligomycin, respiration declined to the resting or leak-compensating state (LEAK state). Uncoupling with stepwise titration to an optimal concentration of the protonophore carbonyl cyanide p-(trifluoromethoxy) phenylhydrazone, FCCP, induced maximal noncoupled respiration as a measure of electron transfer system capacity (ET state).

### Statistical analysis

GraphPad Prism Software was used for all statistical tests. A Student’s *t*-test was used for comparisons between two groups. For comparisons between greater than two groups with one variable, one-way ANOVA followed by a Dunnett’s (compared to one control group) or Tukey’s (comparing all groups to each other) multiple comparisons tests were performed. A two-way ANOVA was used when two variables were present. All data are presented as $\pm$ standard error of the mean (SEM). Experimental replicates are indicated and were performed at least three times. Statistical significance: *P < 0.05, **P < 0.01, ***P < 0.001, ****P < 0.0001.

## Supplementary information

Supplementary Figure 1.

## Data Availability

The datasets generated and/or analysed during the current study are available from the corresponding author on reasonable request.
