# Supplementary material for: BAD sensitizes breast cancer cells to docetaxel with increased mitotic arrest and necroptosis
Source: Sci Rep. 2020 Jan 15;10:355. doi: 10.1038/s41598-019-57282-1 (PMC6962214; doi:10.1038/s41598-019-57282-1)
Supplement: Supplementary file 1 — Supplementary Figure 1. [file 41598_2019_57282_MOESM1_ESM.pdf]

BAD sensitizes breast cancer cells to docetaxel with increased mitotic arrest and necroptosis

Jasdeep Mann[a], Ning Yang[a], Rachel Montpetit[a], Raven Kirschenman[a], Helene Lemieux,[b,c] *Ing Swie Goping[a,d]

Departments of [a]Biochemistry, [b]Medicine, and [d]Oncology, University of Alberta, Edmonton, Alberta, Canada, T6G 2H7; [c]Faculty Saint-Jean, University of Alberta, Edmonton, Alberta, Canada, T6G 2H7

Corresponding Author: Ing Swie Goping, Department of Biochemistry, Faculty of Medicine and Dentistry, University of Alberta, Edmonton, Alberta, Canada T6G 2H7; Tel: 780-492-6130; fax: 780-492-0886; e-mail: igoping@ualberta.ca

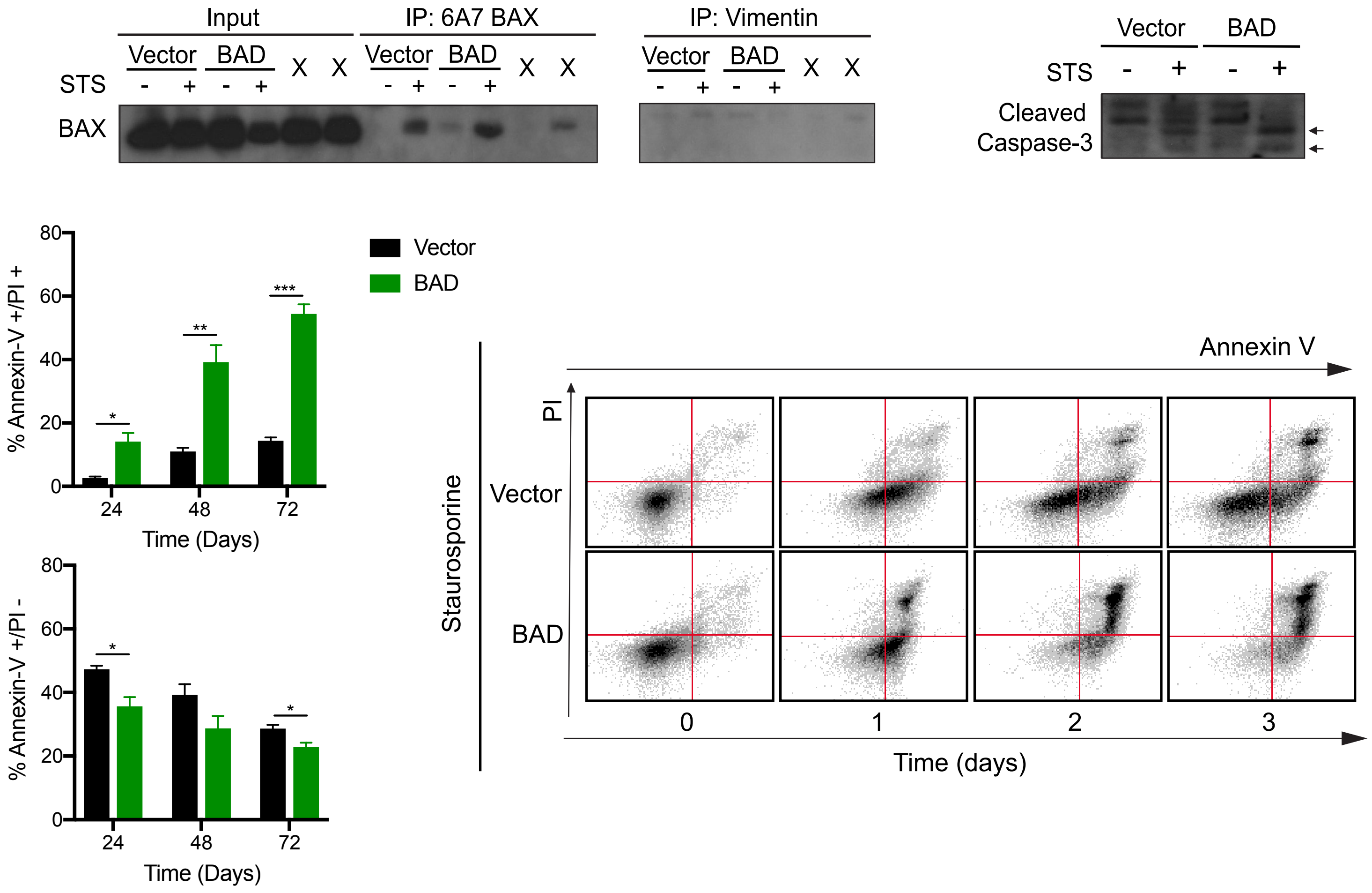

Supplementary Figure 1. BAD increases apoptotic cell death in staurosporine treated cells
Top, left: MDA-MB-231 cells expressing vector or BAD were treated with 2.5 μM staurosporine for 18 hours. Cells were lysed and immunoprecipitated with 6A7 BAX antibody and immunoblotted against BAX protein. Vimentin was used as a negative IP control. Top, right: Input lanes were probed with cleaved caspase-3 antibody. Bottom, left: Cells were treated with 2.5 μM staurosporine for 3 days and stained with Annexin V-647 and PI daily and analyzed via flow cytometry. The Annexin V+/PI+ population (top) and the Annexin V+/PI- population (bottom) are represented in a bar graph. Student's *t*-test; n = 3. Bottom, right: Dot plots from flow cytometric analysis. Annexin V positive cells are on the x-axis, PI positive cells are on the y-axis. Time, in days, is increasing to the right.
